# Impact of sociodemographic factors and screening, diagnosis, and treatment strategies on colorectal cancer mortality in Brazil: A 20-year ecological study

**DOI:** 10.1371/journal.pone.0274572

**Published:** 2022-09-15

**Authors:** Ananda Quaresma Nascimento, Diego Bessa Dantas, Giovana Salomão Melo, Fabiana de Campos Gomes, João Simão de Melo Neto

**Affiliations:** 1 Institute of Health Sciences, Federal University of Pará (UFPA), Belém, PA, Brazil; 2 Medical School Faceres (FACERES), São José do Rio Preto, São Paulo, Brazil; Texas Tech University Health Science, Lubbock, UNITED STATES

## Abstract

Colorectal cancer (CRC) caused 261,060 deaths in Brazil over a 20-year period, with a tendency to increase over time. This study aimed to verify the sociodemographic factors predicting higher mortality caused by CRC and survival rates. Moreover, we aimed to verify whether the performance of screening, diagnostic and treatment procedures had an impact on mortality. Ecological observational study of mortality due to CRC was conducted in Brazil from 2000–2019. The adjustment variable was age, which was used to calculate the age-standardized mortality rate (ASMR). The exposure variables were number of deaths and ASMR. Outcome variables were age-period-cohort, race classification, marital status, geographic region, and screening, diagnostic, and treatment procedures. Age-period-cohort analysis was performed. ANOVA and Kruskal-Wallis test with post hoc tests were used to assess differences in race classification, marital status, and geographic region. Multinomial logistic regression was used to test for interaction among sociodemographic factors. Survival analysis included Kaplan-Meier plot and Cox regression analysis were performed. Multivariate linear regression was used to test prediction using screening, diagnosis, and treatment procedures. In Brazil, mortality from CRC increased after age 45 years. The highest adjusted mortality rates were found among white individuals and in the South of the country (p < 0.05). Single, married, and widowed northern and northeastern persons had a higher risk of death than legally separated southern persons (p < 0.05). Lower survival rates were observed in brown and legally separated individuals and residents from the North (p < 0.05). An increase in first-line chemotherapy and a decrease in second-line chemotherapy were associated with high mortality in the north (p<0.05). In the south, second-line chemotherapy and abdominoperineal rectal resection were associated with high mortality (p < 0.05). Regional differences in sociodemographic factors and clinical procedures can serve as guidelines for adjusting public health policies.

## Introduction

Colorectal cancer (CRC) is defined by the International Classification of Diseases and Related Health Problems (ICD-10), as a cancer of the colon, rectosigmoid junction, or rectum [1]. CRC has become a dominant type of neoplasm in several countries, accounting for about 10% of cancer-related mortality worldwide [2]. According to recent statistics from the International Agency for Research on Cancer, CRC is the third most common malignant disease in the world, with 1.85 million new cases per year [3]. In Brazil, it is estimated that in the triennium from 2020 to 2022 inclusive, 20,540 men and 20,470 women will be diagnosed with CRC per year [4].

The increase in incidence is related to genetic and environmental factors associated with lifestyle [5]. Therefore, sociodemographic variables have a considerable impact on various types of cancer, whether through differences in access to health services or through delays in receiving treatment. These factors influence the prognosis of the condition and are directly related to the increase in mortality [6]. However, these variables need to be better understood in relation to local characteristics. Thus, we studied these factors in the Brazilian national scenario for the first time, which is fundamental due to the territorial extension and because each region has very specific characteristics.

In addition, understanding the impact of screening and diagnostic procedures on mortality is necessary. Therefore, when CRC is diagnosed at an early stage, the 5-year survival rate is close to 90%. In contrast, due to the protracted and silent nature of the disease and hence a greater predilection for the occurrence of metastases, when CRC is diagnosed late, this percentage reaches 13% [7]. Recent advances in the screening process for early detection, including timely examination using colonoscopy, blood and fecal tests, and computed tomography, have been developed [8]. Studies analyzing the impact of these resources in Brazil are still needed. Although there have been evident improvements in the screening and diagnosis of CRC, participation in routine screening remains low. This low adherence, particularly among individuals with known risk factors or a family history of the disease is more evident in countries with a private health system or among populations with a lower socioeconomic status [9].

The primary treatment for CRC is surgical resection of the tumor and affected tissues [10, 11]. However, in cases of late diagnosis, where metastases have already occurred, the prognosis is worse even if surgical resection is performed [12]. But, other treatment options, including chemotherapy, have reduced the mortality of CRC in developed countries, even in the face of increasing incidence [13]. Considering that Brazil is a developing country with specific regional characteristics, understanding the response to different types of treatment is necessary to adapt public policies and the organization of the health system.

Thus, this study aimed to verify the social and demographic factors that predict higher CRC mortality and worse survival rates. In addition, we aimed to verify whether the performance of screening, diagnostic, and treatment procedures impacted mortality reduction. Initially, our hypothesis was that elderly individuals (> 60 years old), individuals undergoing periods of epidemiological transition, individuals from less developed regions (the North and Northeast of Brazil), and individuals from vulnerable social categories (racial classification [black] and marital status [single or widowed]) would have higher mortality rates and lower survival rates. Our second hypothesis was that there would be a negative relationship between CRC mortality and the frequency of screening, diagnostic, and treatment procedures performed.

## Materials and methods

### Ethical aspects

In this study, secondary data on CRC mortality available on public domain databases were analyzed. Therefore, approval by the Research Ethics Committee was not required, as per the guidelines of the National Health Council 510 (04 July 2016) [14].

### Type of study

An observational study with an ecological design [15].

### Population

Secondary data of individuals of both sexes, who had died due to CRC between 2000 and 2019 and were registered in the Health Information System of the Ministry of Health of Brazil, were reviewed for inclusion in this study.

### Inclusion and exclusion criteria

Patients who died between the years 2000 and 2019 and were classified with ICD-10 codes C18 (malignant neoplasm of the colon), C19 (malignant neoplasm of the rectosigmoid junction), or C20 (malignant neoplasm of the rectum), were included [16]. Information on outpatient and hospital procedures that had been recorded from 2008 were also reviewed.

Deaths registered outside the study period and individuals with missing data for the variables analyzed were excluded from the analysis.

#### Database

The Department of Informatics of the Unified Health System (DATASUS) of the Brazilian Ministry of Health platform is an open-access secondary database. Information on mortality from CRC; sociodemographic variables; and screening, diagnostic, and treatment procedures was obtained from the DATASUS in the registers of the Mortality Information System (SIM), Hospital Information System (SIH), and Outpatient Information System (SIA). Information registered in the SIH and SIA was analyzed from 2008 [17]. The units of analysis investigated were the five geographic regions of Brazil according to the Brazilian Institute of Geography and Statistics (IBGE).

The SIM was created in 1975 and refers to a national epidemiological surveillance system of death records in all Brazilian cities based on the certificate of the cause of death, entry of which is mandatory throughout the national territory [17, 18]. The vital registration data quality of Brazil is high, with a usability of approximately 73%-84% and completeness of 100% [19]. However, the percentage of coded deaths that had ill-defined causes was 10.14% in 2015 in Brazil [20].

In addition, population quantity data were obtained from the IBGE [21]. The world standard population was used to adjust variables by age according to the World Health Organization [22].

### Variables analyzed

Initially, population quantity data for calculation of the specific crude mortality rate (CMR) were obtained from a projection using population data from the IBGE [21]. The number of deaths used to calculate the CMR was obtained according to the place of residence. CMR was calculated by dividing the number of deaths in a given time period for a given population by the total population [22]. The adjustment variable was the age at death, which was used to calculate the age-standardized mortality rate (ASMR). The world standard population was used to calculate the ASMR [23]. Subsequently, the ASMR was estimated using the indirect method compared to the world standard population per 100,000 inhabitants. Therefore, the exposure variables were the number of deaths and the ASMR.

The outcome variables were age-period-cohort; racial classification; marital status; geographic region; and screening, diagnostic, and treatment procedures.

The social variables analyzed were age, period of birth, year of death (cohort), racial classification according to skin color (white, black, yellow, brown, or indigenous), and marital status (single, married, widowed, or legally separated). The IBGE classification states that race/ethnicity is verified by self-declaration. Furthermore, the differences between groups in terms of ethnic, linguistic, cultural, or historical characteristics were not well established; thus, any attempt to classify people according to other categories, such as Caucasian or Hispanic, could be inaccurate in Brazil. The demographic variables were geographic regions (North, Northeast, Southeast, South, and Midwest).

Data on the following outpatient procedures were collected: screening and diagnosis (fecal occult blood test, rectosigmoidoscopy, and colonoscopy) and treatment (colon adenocarcinoma chemotherapy, advanced colon adenocarcinoma chemotherapy [first line], advanced colon adenocarcinoma chemotherapy [second line], advanced rectal adenocarcinoma chemotherapy [first line], and advanced rectal adenocarcinoma chemotherapy [second line]). Data on the following hospital treatment procedures were collected: local excision of a rectal tumor in oncology, abdominoperineal resection of the rectum in oncology, abdominal rectosigmoidectomy in oncology, complete abdominoperineal resection of the rectum in oncology, abdominal rectosigmoidectomy in oncology, abdominal resection of the complete abdominoperineal rectum, and abdominoperineal rectosigmoidectomy.

### Statistical analysis

To analyze the age-period-cohort (APC) regression model, the APC Web Tool (Biostatistics Branch, National Cancer Institute, Bethesda, MD, USA) was used [24]. The APC model includes parameters (trends and deviations) that describe the mathematical relationships between the cancer rate and attained age, calendar period (year of diagnosis), and birth cohort (year of birth) [24]. The number of deaths and the population at risk were grouped at regular 5-year intervals to limit the estimated parameters. A total of 18 age groups (from 0–4 years to 75–89 years), 4 periods (from 2000–2004 to 2015–2019), and 21 birth cohorts of 5 years each (1915 to 2015) were included. Differences were considered statistically significant at p < 0.05 for the Wald test (hypothesis test). The following functions were estimated for all variables analyzed: net deviations (global annual percentage change according to the calendar period and birth cohort), local deviations (annual percentage changes for each age group according to the calendar period and birth cohort), all age deviations (the adjusted longitudinal and transverse age curves are log-linear), all period deviations (adjusted time trends and period rates are log-linear), all cohort deviations (cohort rates are log-linear, and all local deviations are equal to net deviations), and all period (or cohort) rate ratios (RR; age incidence pattern in each period [or cohort]). This model estimates the contribution of the effects of age, period, and birth cohort on mortality from CRC.

The results of descriptive analysis were expressed as a measure of the central tendency and dispersion. To verify normality of the data, the Kolmogorov–Smirnov or Shapiro–Wilk tests were performed. To determine whether there were statistically significant differences between the means or medians of the ASMR in terms of racial classification, marital status, and geographic region, one-way analysis of variance (parametric) and the Kruskal–Wallis test (non-parametric) with Tukey’s and Dunn’s post hoc tests, respectively, were used.

Multinomial logistic regression analysis was used to investigate the effects of the interaction between social and demographic factors on mortality in Brazil. Reference categories were defined based on the highest ASMR values between groups. However, in relation to racial classification, data from indigenous individuals were excluded from the analysis owing to limitations and the occurrence of failures in the notification of deaths in this population [25]. Odds ratios (ORs) with 95% confidence intervals (CIs) were used to quantify the degree of association.

For survival analysis, we used the microdatasus package [26] to obtain the survival time considering the period from birth to death according to racial classification, marital status, and geographic region. To estimate survival, Kaplan-Meier curves were constructed for racial classifications, marital status, and geographic regions, and the log-rank, Breslow, and Tarone-Ware tests were used to compare the survival rate distributions along the curve for the different groups. The variables that presented a statistically significant p-value were included in Cox’s proportional hazards model for survival time to investigate whether there was an association between survival time and the different variable categories. Univariate models were also used. The data violated the proportionality assumption of the hazard ratio, but we considered that the p-value was dependent on the sample size, and a large sample size would produce a high significance with a minimal violation of the assumption, as reported by In and Lee [27].

Multivariate linear regression analysis was used to verify the prediction of mortality using screening, diagnostic, and treatment procedures for CRC in Brazil and in separate geographic regions.

Statistical significance was set at p < 0.05. SPSS Version 26.0 (IBM Corp. Released 2019. IBM SPSS Statistics for Windows, version 26.0. Armonk, NY: IBM Corp.) and RStudio Team (2021) (RStudio: Integrated Development Environment for R) were used for statistical analyses.

## Results

Between 2000 and 2019, 261,060 cases of CRC-associated mortality in Brazil were registered in the public health system. Of these, 134,632 females (51.5%; 9.9/100,000 inhabitants) and 126,399 males (48.4%; 6.4/100,000 inhabitants) died.

### Social factors

#### Age-period-cohort effect

**Fig 1:**
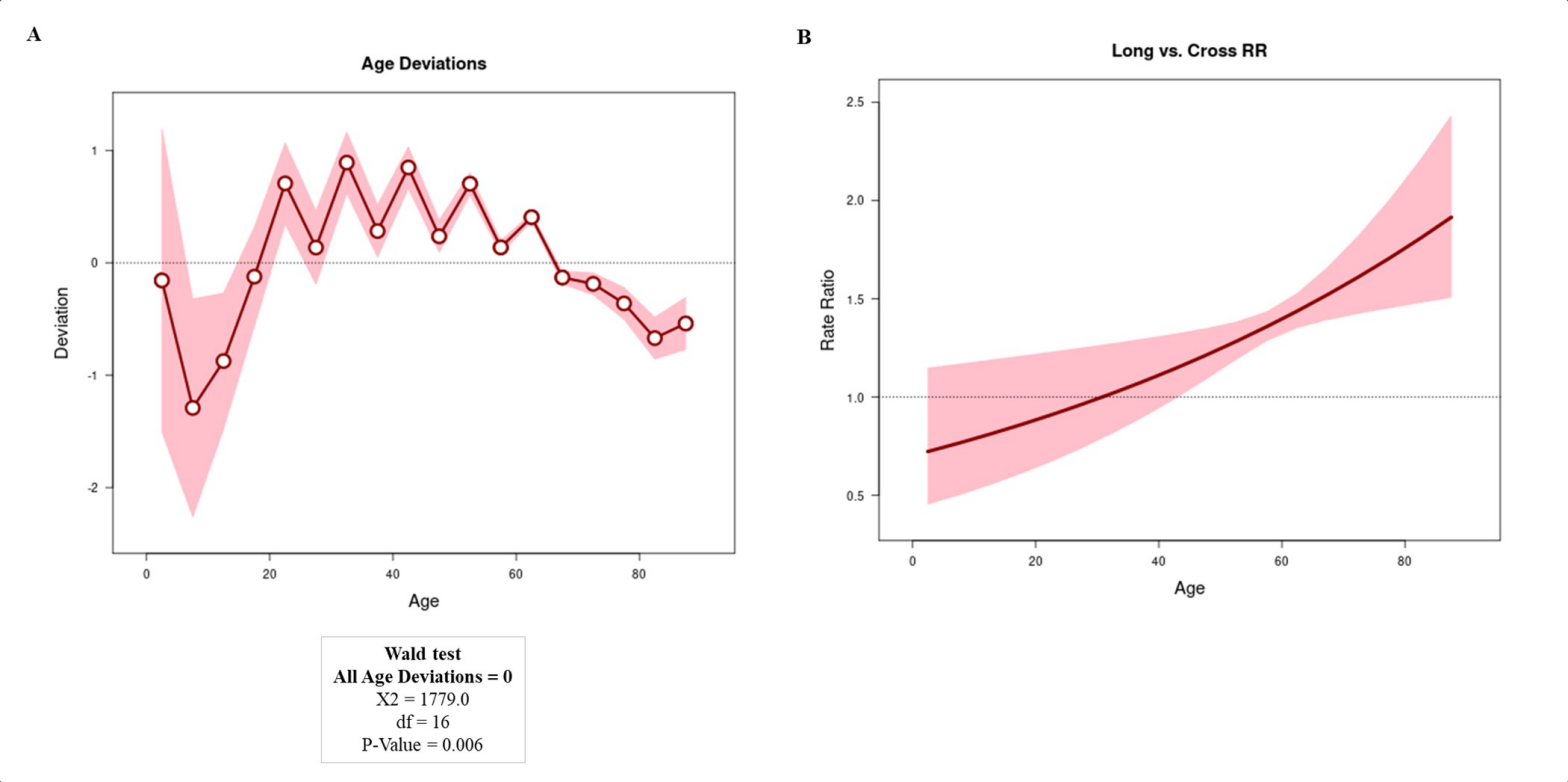
Age-period-cohort analysis using the Wald test (A), with the analysis of all age deviations (A) and the mortality rates (B) by age. In Fig 1A and 1B, the lines represent the oscillation of the Y-axis value in relation to the X-axis; the circle represents the moment on the X-axis; the shadows above the lines represent the 95% CIs.

**Fig 2:**
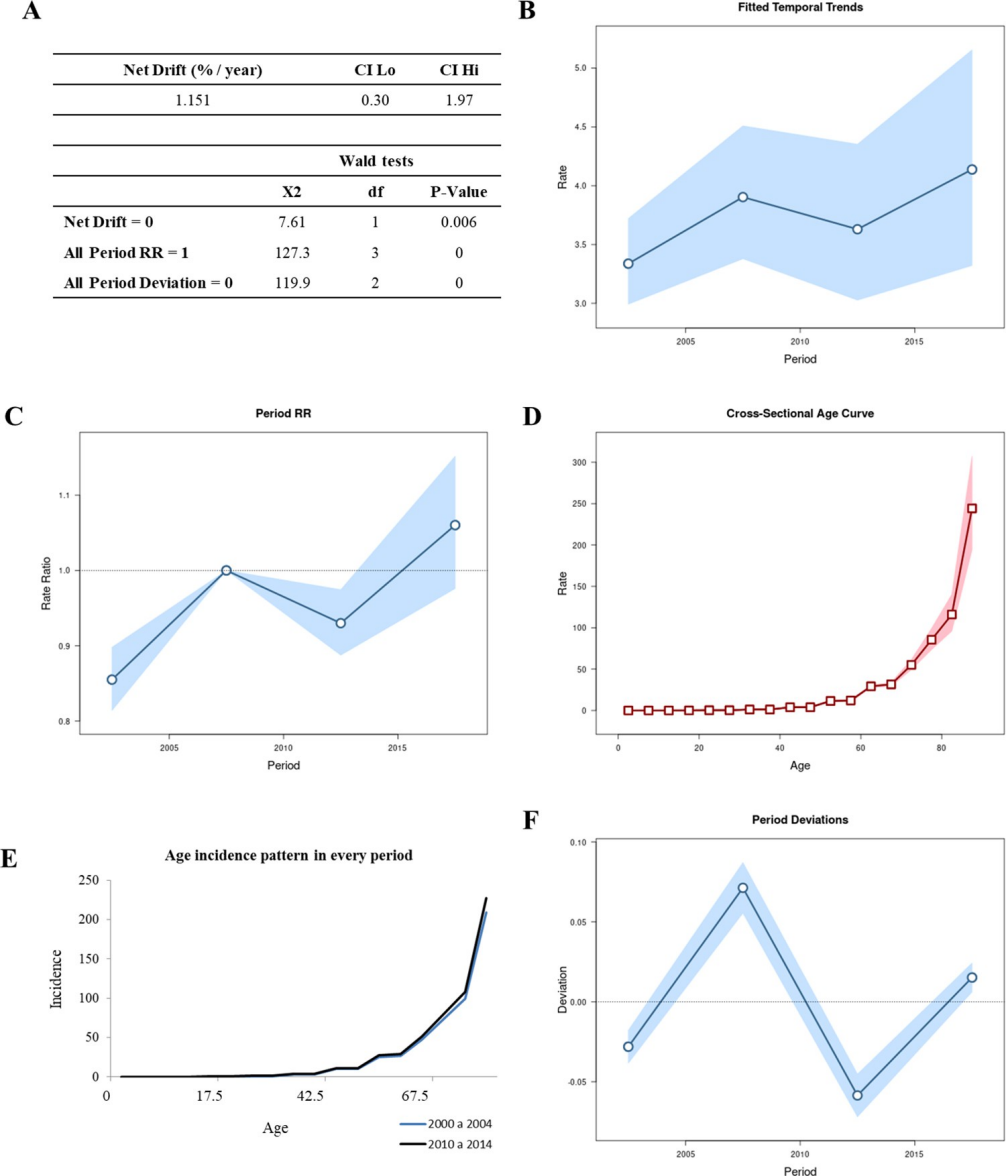
Age-period-cohort analysis using the Wald test, with the analysis of net drift (A), temporal trends (B), period rate ratios (C), the cross-sectional age curve (D), the age incidence pattern for every period (E), and all period deviations (F). In Fig 2B-2D and 2F the lines represent the oscillation of the Y-axis value in relation to the X-axis; the circle or square represents the moment on the X-axis; the shadows above the lines represent the 95% CIs.

**Fig 3:**
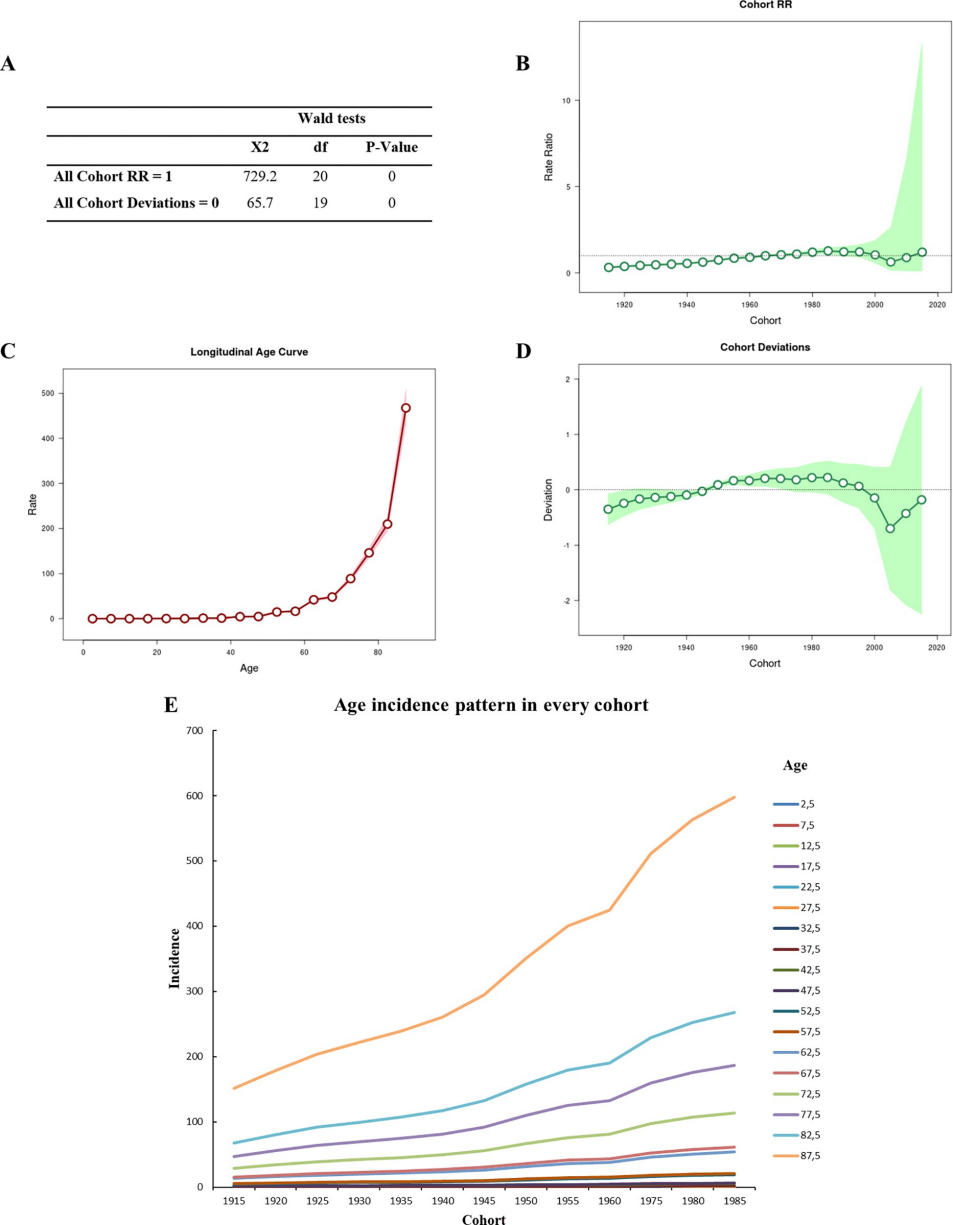
Age-period-cohort analysis using the Wald test (A), with the cohort RR analysis (B), the longitudinal age curve (C), cohort deviations (D), and age incidence patterns for every cohort (E). In Fig 3B-3D, the lines represent the oscillation of the Y-axis value in relation to the X-axis; the circle represents the moment on the X-axis; the shadows above the lines represent the 95% CIs.

Figs 1–3 show the results obtained from the APC analysis. Fig 1A presents the age deviation, with the linear trend in age calculated based on non-linear age effects. We observed that the age curves were linear, with an increased progressive risk after the age of 45 years (RR 1.21; 95% CIs: 1.08–1.34) (Fig 1B).

During the period 2000–2019, the annual percentage change in the expected age-adjusted rates (net drift), was 1.15% per year (Fig 2A). The expected rates over time in accordance with the reference age group adjusted for cohort effects during the respective period increased at a constant rate, as shown in Fig 2B. The age incidence pattern for every period is presented in Fig 2E, calculated from the period RR (Fig 2C) and cross-sectional age curve (Fig 2D), with significant intervals from 2000 to 2004 and from 2010 to 2014. Fig 2F presents the period deviation, with temporal trends and period rate ratios increasing linearly with age, independent of non-linear period effects (from 2005 to 2009 and from 2015 to 2019).

The proportion of age-specific mortality rates for each birth cohort shows a pattern of increasing incidence with age over time, particularly for the significant intervals from 1915 to 1960 and from 1975 to 1985 based on the cohort RR calculation (Fig 3B) and the longitudinal age curve (Fig 3C), as shown in Fig 3E. The cohort deviations were significant for the birth cohorts from 1915 to 1920, 1935 to 1940, and 1950 to 1970 (Fig 3D).

#### Age-adjusted mortality rates

**Fig 4:**
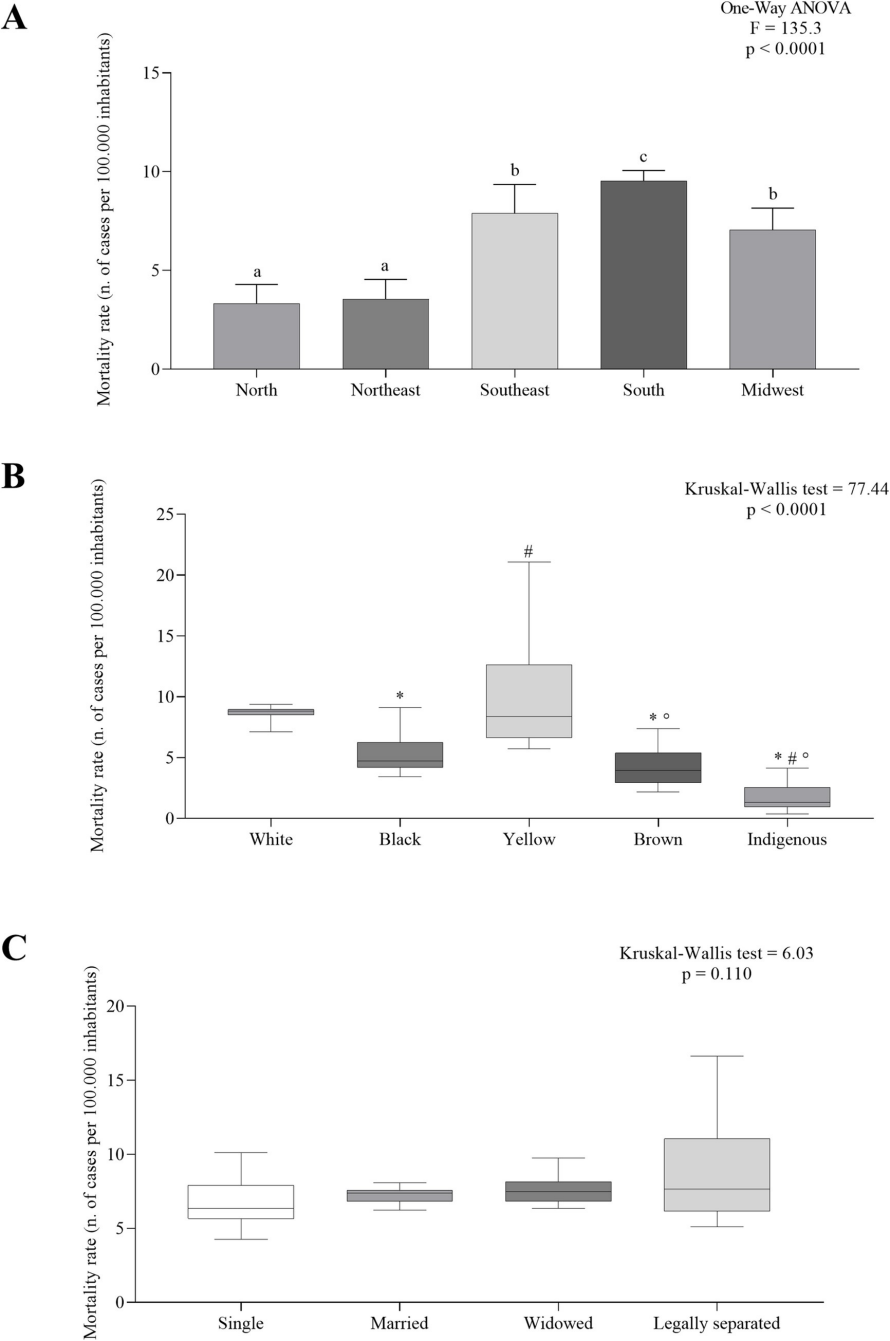
Colorectal cancer mortality rates according to geographic region (A), race (B), and marital status (C). ^a, b, c^ p < 0.05, post-hoc Bonferroni. *p < 0.05, post-hoc Bonferroni in comparison with White. #p < 0.05, post-hoc Bonferroni in comparison Black.°p < 0.05, post-hoc Bonferroni in comparison with Yellow.

Fig 4 shows a comparison of age-adjusted mortality rates across racial classification, marital status, and geographic region.

In Brazil, white (9.16 [7.14–10.93]) and yellow (6.40 [5.70–16.39]) individuals had a higher age-adjusted mortality rate (Fig 4A). However, brown (3.94 [2.16–5.98]) and indigenous (1.32 [0.37–4.13]) individuals had a lower age-adjusted mortality rate (Fig 4A). There were no statistically significant differences between groups according to marital status (Fig 4B).

### Demographic factors

#### Age-adjusted mortality rates

Regarding demographics, the South had the highest age-adjusted mortality rate (9.5 ± 0.52), while the North (3.31 ± 0.97) and the Northeast (3.55 ± 0.98) had the lowest age-adjusted mortality rates (Fig 4C).

### Interaction between social and demographic factors

Table 1 shows the social and demographic factors predicting mortality in Brazil using multinomial logistic regression. In almost all geographic regions, individuals racially classified as black, yellow, and brown had a lower mortality rate than those racially classified as white, except individuals racially classified as brown from the North, who had a higher mortality rate than those from the South. Single, married, and widowed individuals from the North and Northeast had a higher mortality rate than legally separated individuals from the South. Married individuals from the Midwest and widowed individuals from the Southeast and Midwest had a lower mortality than legally separated individuals from the South.

**Table 1:** Sociodemographic factors that predict mortality in Brazil in relation to the largest category.

	Racial classification (White reference)			Marital status (Legally separated reference)		
Regions (South reference)	Black OR (CI 95%) *p*	Yellow OR (CI 95%) *p*	Brown OR (CI 95%) *P*	Single OR (CI 95%) *p*	Married OR (CI 95%) *p*	Widowed OR (CI 95%) *P*
**North**	0.12 (0.11–0.14) < 0.0001*	0.07 (0.05–0.10) < 0.0001*	0.84 (0.50–1.40) 0.513	2.46 (2.00–3.03) < 0.0001*	1.91 (1.57–2.33) < 0.0001*	1.38 (1.12–1.70) 0.002*
**Northeast**	0.20 (0.19–0.22) < 0.0001*	0.05 (0.04–0.06) < 0.0001*	0.25 (0.15–0.41) < 0.0001*	2.23 (1.91–2.61) < 0.0001*	1.73 (1.50–2.00) < 0.0001*	1.40 (1.20–1.64) < 0.0001*
**Southeast**	0.54 (0.50–0.58) < 0.0001*	0.77 (0.68–0.87) < 0.0001*	0.21 (0.13–0.34) < 0.0001*	1.06 (0.92–1.22) 0.379	0.91 (0.80–1.03) 0.168	0.85 (0.74–0.97) 0.022*
**Midwest**	0.21 (0.19–0.23) < 0.0001*	0.18 (0.14–0.22) < 0.0001*	0.37 (0.21–0.67) 0.001*	0.90 (0.77–1.07) 0.256	0.85 (0.72–0.99) 0.037*	0.65 (0.55–0.76) < 0.0001*

* p < 0.05, Multinomial Logistic Regression with main effects.

### Survival

**Fig 5:**
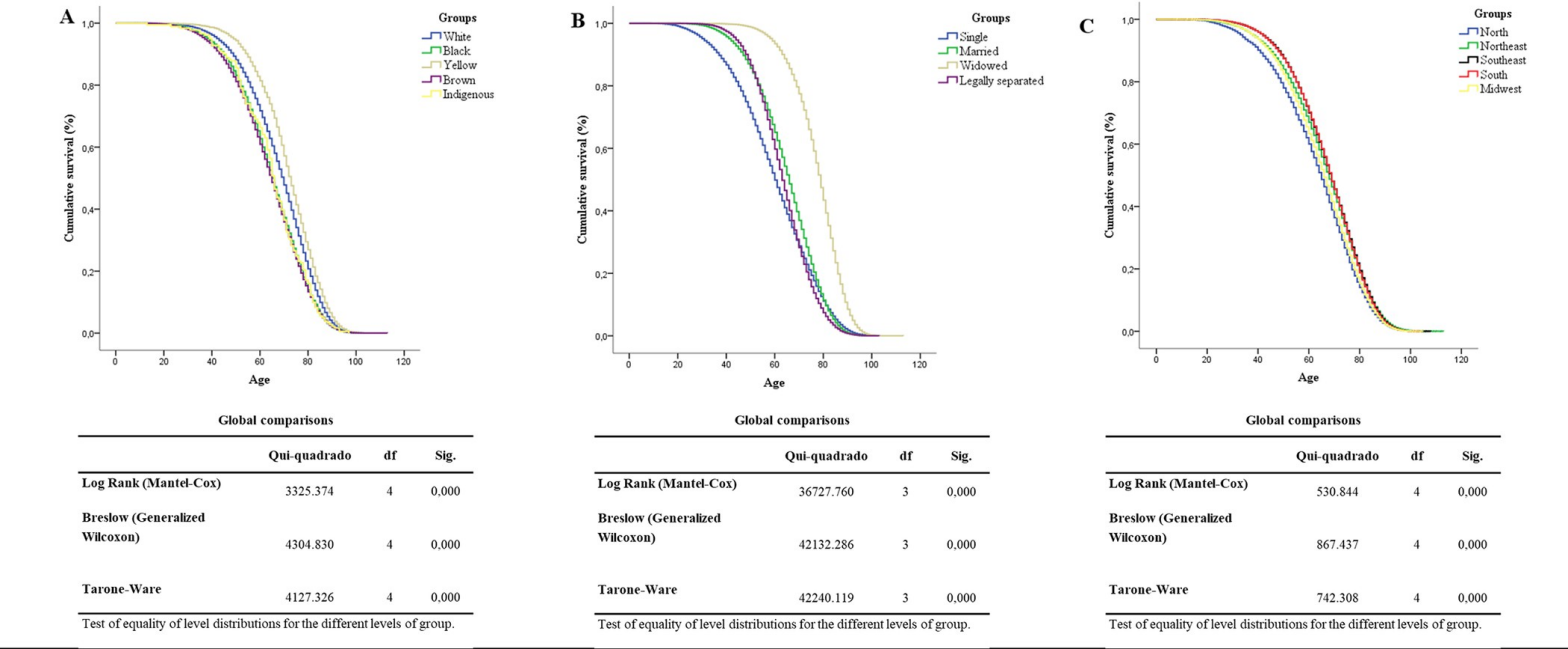
Survival curves among the different racial classification groups (A), marital status (B) and geographic regions (C) using Kaplan-Meier plots.

Fig 5 shows the Kaplan–Meier curves according to racial classification (Fig 5A), marital status (Fig 5B), and geographic region (Fig 5C), with statistical significance along the entire curve. Table 2 presents the Cox regression analysis with the reference categories for each variable. Regarding racial classification, individuals classified as white had lower survival rates than those classified as black, brown, or indigenous; only those classified as yellow showed higher survival rates. Single, married, and widowed individuals had higher survival rates than legally separated individuals. Individuals from the North and Midwest had lower survival rates than those from the South. Individuals from the Southeast had a higher survival rate than those from the South.

**Table 2:** Colorectal cancer survival rates according to geographic region, racial classification and marital status in Brazil between 2000 and 2019 using Cox regression univariate analyses, in relation to the largest category of mortality.

	*p*	HR	95% CI
**Race** (White reference)			
Black	< 0.0001	1.248	1.226 to 1.270
Yellow	< 0.0001	0.829	0.800 to 0.860
Brown	< 0.0001	1.288	1.276 to 1.301
Indigenous	0.004	1.238	1.070 to 1.432
**Marital status** (Legally separated reference)			
Single	< 0.0001	0.952	0.935 to 0.969
Married	< 0.0001	0.839	0.825 to 0.852
Widowed	< 0.0001	0.367	0.361 to 0.373
**Regions** (South reference)			
North	< 0.0001	1.208	1.177 to 1.240
Northeast	0.840	1.001	0.987 to 1.016
Southeast	0.002	0.984	0.974 to 0.994
Midwest	< 0.0001	1.135	1.114 to 1.156

Hazard ratio (HR). 95% Confidence Interval (95%CI).

### Prediction of mortality by performing screening, diagnostic, and treatment procedures

Table 3 shows the prediction of mortality in Brazil and in different geographic regions by performing screening, diagnosis and treatment procedures for CRC. No screening or diagnostic test was associated with mortality at the national or geographic level. When analyzing colon and rectal chemotherapies, increases in first-line chemotherapy and decreases in second-line chemotherapy were associated with increased mortality in the Northern region. In the southern region, second-line chemotherapy accompanied the highest mortality rate, while, surgically, abdominoperineal resection of the rectum in oncology was associated with increased mortality.

**Table 3:** Prediction of mortality in Brazil and different geographic regions by performing screening, diagnosis and treatment procedures for colorectal cancer.

	Geographic region					
	Brazil	North	Northeast	Southeast	South	Midwest
**Screening and diagnostic procedures**						
Fecal occult blood test	β = -0.175	β = 0.561	β = 0.287	β = 7.999	β = -2.091	β = 0.032
	t = -1.070	t = 2.538	t = 0.503	t = 1.298	t = -0.387	t = 0.077
	p = 0.290	p = 0.085	p = 0.650	p = 0.285	p = 0.128	p = 0.944
Rectosigmoidoscopy	β = -0.162	β = -0.060	β = 0.244	β = -0.636	β = -0.347	β = -0.088
	t = -1.164	t = -0.169	t = 0.447	t = -0.503	t = -0.387	t = -0.114
	p = 0.250	p = 0.876	p = 0.685	p = 0.649	p = 0.724	p = 0.916
Colonoscopy	β = 0.544	β = 0.112	β = -0.369	β = 3.914	β = 2.248	β = 0.678
	t = 1.820	t = 0.497	t = -0.142	t = 0.865	t = 0.638	t = 1.617
	p = 0.075	p = 0.654	p = 0.896	p = 0.451	p = 0.569	p = 0.204
**Chemotherapy treatment**						
First line chemotherapy for the colon and rectum	β = -0.423	β = 2.444	β = 1.209	β = -8.887	β = 10.540	β = 0.288
	t = -1.373	t = 5.453	t = 0.450	t = -1.546	t = 2.719	t = 0.423
	p = 0.194	p = 0.012*	p = 0.684	p = 0.220	p = 0.073	p = 0.701
Second line chemotherapy for the colon and rectum	β = 0.378	β = -1.384	β = 0.010	β = -1.003	β = 2.034	β = 0.194
	t = 1.316	t = -4.101	t = 0.011	t = -0.985	t = 5.019	t = 0.642
	p = 0.194	p = 0.026*	p = 0.992	p = 0.397	p = 0.015*	p = 0.566
**Surgical treatments in oncology**						
Excision of rectal tumor in oncology	β = 0.051	β = 0.814	β = -0.195	β = -0.740	β = -1.762	β = -0.148
	t = 0.269	t = 1.568	t = -0.592	t = -0.506	t = -1.810	t = -0.577
	p = 0.789	p = 0.215	p = 0.595	p = 0.648	p = 0.168	p = 0.604
Abdominoperineal rectum resection in oncology	β = 0.127	β = -0.901	β = 0.089	β = -0.523	β = 2.091	β = -0.060
	t = 0.462	t = -2.635	t = 0.346	t = -0.739	t = 4.773	t = 0.213
	p = 0.646	p = 0.078	p = 0.752	p = 0.513	p = 0.017*	p = 0.845
Abdominal rectosigmoidectomy in oncology	β = 0.350	β = 0.087	β = 0.249	β = -3.092	β = -0.179	β = 0.102
	t = 1.005	t = 0.144	t = 0.664	t = -1.793	t = -0.563	t = 0.440
	p = 0.319	p = 0.895	p = 0.554	p = 0.171	p = 0.613	p = 0.690

Standardized coefficient of linear regression (β). t-Statistic: “t” refers to observed t.

* p < 0.05, Multivariate linear regression analysis.

## Discussion

CRC is a slowly progressing neoplasm that is highly curable if detected early [28]. In Brazil, regional disparities contribute to different distribution patterns of mortality [29]. We observed in this study that sociodemographic factors and access to screening, diagnosis, and treatment strategies impacted this pattern over a 20-year period.

Age is one of the most important risk factors in the development of CRC [5]. In this study, an increasing trend of mortality was observed in those aged > 45 years, with the risk increasing by 1.15% per year. This contributes to the increase in mortality rates and the worsening of prognosis over time. The increase in mortality in individuals aged < 50 years is related to the advanced stage of disease, with associated factors including predisposition, low patient awareness, and low adherence to screening, and consequently, more patients are diagnosed with CRC at an advanced stage [30]. Therefore, Brazilians with risk factors should initiate preventive measures before the age of 45 years.

Studies have shown increasing trends in CRC mortality in specific states [29, 31, 32], the capital [33], and in Brazil as a whole in other years, until 2017 [28, 34, 35]. However, we did not find consistent studies that extended the analysis to 2019 at the national level; therefore, there is a gap in this period. We observed significant mortality rates between certain specific periods (2000–2004 and 2010–2014). Both these periods involved important epidemiological transitions, possibly due to ongoing economic and social changes related to increased exposure to risk factors and modifications of dietary patterns [35], such as a high intake of red and processed meats, refined grains, starches, and sugars [36]. In addition, the economic recession that occurred in the country from 2012 to 2017 was associated with > 30,000 deaths due to unemployment and worsening socioeconomic conditions. There was a dramatic increase in some of the causes of death with cancer being the leading one [37].

Individuals racially classified as white had the highest adjusted mortality rate. These results could be attributed to genetic, environmental, and social factors. According to several studies, inflammatory bowel disease (IBD) is highly prevalent among Caucasians [38, 39], and they are at a higher risk of developing CRC [40–43]. This increased risk is due to the presence of genetic polymorphisms in people of European ancestry [44] as well as other molecular changes that differentiate it from sporadic CRC [42, 45, 46]. Additionally, environmental risk factors that contribute to the development of CRC include the adoption of habits seen in Western countries such as inadequate nutrition [47], physical inactivity [48], high alcohol consumption [49], smoking [50], and obesity [51].

In addition, people from the South had the highest adjusted mortality rate. This is one of the regions in the country with a large number of individuals suffering from IBD [38]. In addition, the process of colonization in the South region, primarily by European immigrants, contributed to Brazil having the fifth largest European genetic lineage in Latin America [52], with a high predominance of white individuals of European ancestry, explaining the high mortality rate. Another factor that may enhance the characteristics of racial classification in this region is the better socioeconomic situation [53–55]. The urbanization that accompanied the industrialization of the South contributed to the improvement of these conditions. However, it also led to a greater exposure to risk factors for CRC, such as greater availability and consumption of foods rich in carbohydrates, proteins, total fat, trans fat, cholesterol, saturated fatty acids, and iron [56]; a more sedentary lifestyle; decreased physical activity; and increased prevalence of obesity [57].

Individuals from the North who were brown and legally separated had lower survival rates. The proportion of brown individuals in the population is the highest in the North [21]. This is due to the high degree of miscegenation resulting from the colonization process, which greatly contributes to Indo-American genetic ancestry in this population [58]. This ancestry has been associated with the mucinous histological subtype of CRC [52], which is associated with characterized by an impaired response to neoadjuvant chemoradiotherapy and a worse overall survival [59]. The worst prognosis associated with this oncological subtype is related to the presence of larger tumors diagnosed at more advanced stages with high rates of metastasis [60, 61]. This leads to a poor response to chemotherapy and reduced survival compared with patients with non-mucinous CRC [62]. These findings help explain the lower survival rate of individuals racially classified as brown.

In terms of marital status, there were no significant differences between the groups in the Brazilian population. However, legally separated individuals had lower survival rates. Similarly, in the USA, divorced or separated individuals were less likely to adhere to the CCR screening guidelines than individuals who were married or single [63]. Matrimony is an important convention in society that provides mutual emotional support, with the spouse playing a crucial role in health-related monitoring and care [64]. However, single, married, and widowed individuals from the North and Northeast have a higher risk of dying. In this sense, consideration must be given to the greater time interval between diagnosis and treatment in these regions. Nevertheless, the lower survival rate among residents of the North can be explained by the longest waiting time for chemotherapy in Brazil [65]. In addition, it is recognized that the greater availability of these services is concentrated in the Southeast and South regions [66].

In the northern region, the highest performance of first-line chemotherapy was associated with increased mortality. It has been shown that initiating CRC treatment takes the longest duration in this region [65]. This had a direct effect on mortality. Nevertheless, a negative association was found between a low number of second-line chemotherapy procedures for CRCs and a high mortality rate, suggesting that public policies in this region need to be changed to reduce this disparity. The northern region includes the Brazilian Amazon, a territory that contains sparsely populated rural areas, with long distances and isolation from urban areas where health services are located [67]. In this region, it is difficult to reach and provide health services, especially because rivers are one of the primary means of interconnection [67], directly impacting the initiation and monitoring of appropriate treatment.

In the southern region, second-line chemotherapy and abdominoperineal resection of the rectum were performed in oncology, but this did not reduce the mortality rate. The severity of histologic inflammation in IBD, which is complicated by earlier and morphologically more aggressive carcinomas [43, 45, 46] in the predominantly white population of European descent, may be a determining factor. Furthermore, second-line chemotherapy options for CRCs have not yet been fully defined [68], It is a generalized, and not a personalized, approach with an uncertain risk-benefit ratio [69], and directly affects mortality. Abdominoperineal resection of the rectum is an extensive procedure involving the excision of a large quantity of tissue and is often performed in patients with a severe clinical state. In this type of resection, the rectum and anal canal are excised, and the anus closed creating a permanent colostomy [70]; this is associated with significant mortality and morbidity [71], which helps explain our findings.

Therefore, sociodemographic factors and disparities in treatment procedures allow us to emphasize the importance of targeted and equitable public health measures to reduce mortality and increase survival from CRC in specific regions of Brazil.

The limitations of this study are the following: use of a secondary database, resulting in ecological bias; lack of data from private services not covered by SUS; and racial classifications being determined by the medical team responsible for completing the death certificate, which raises the possibility of differing self-reported data. Considering the limitations of this study, it is essential to conduct control or cohort case studies. There is the possibility of delays or errors during the insertion of data into the Ministry of Health system, mainly due to connectivity infrastructure disparities in different regions of the country. Finally, we noted that approximately 8% mortality records in the SIM had a non-specific classification (ICD-10: R00-R99) during the study period.

## Conclusion

In the present study, we found that mortality from CRC showed an increasing trend in those aged > 45 years, with an annual increased risk of 1.15% over the 20-year study period, with a constant risk that increased with age and year of birth. Period rate ratios increased during the epidemiological transition. Those living in the South and racially classified as white had the highest adjusted mortality rates. Single, married, and widowed individuals had a higher risk of death than legally separated individuals if they resided in the northern and northeastern regions compared with the South. Brown, legally separated patients living in the northern region had lower survival rates. Increases in first-line chemotherapy and decreases in second-line chemotherapy were associated with increased mortality in the northern region. In the southern region, second-line chemotherapy and abdominoperineal resection of the rectum in oncology were associated with increased mortality.

## List of abbreviations

APC: Age-period-cohort
CRC: Colorectal cancer
DATASUS: Department of Informatics of the Unified Health System
IBGE: Brazilian Institute of Geography and Statistics
ICD-10: International Classification of Diseases and Related Health Problems 10^th^ Revision
SIA: Outpatient Information System
SIH: Hospital Information System
SIM: Mortality Information System
